# The Newer Remedies, Including Synonyms, Sources, Methods of Preparation, Tests, Etc.

**Published:** 1899-06

**Authors:** 


### BOOK NOTICE.

THE NEWER REMEDIES, including synonyms, sources, methods of preparation, tests, etc. A reference manual for physicians, pharmacists, and students. By Virgil Coblentz, A. M., Phar. M., Ph. D., F. C. S., etc., Professor of Chemistry and Physics in the New York College of Pharmacy. Third edition, revised and very much enlarged. Philadelphia: P. Blakiston's Son & Co., 1012 Walnut Street. 1899. Price, $1.00.

This manual of one hundred and fifty pages contains a short description of each of the newer remedies, all arranged in alphabetical order.

The work includes in its list all the famous "coal-tar derivatives," that in bewildering number, have come like a deluge into the materia medica and pharmacology of the old school of medicine.

In a separate chapter entitled "Addenda," it contains a list, with descriptions, of all the various therapeutic agents derived from the organs of animals, such as the thyroid and thymus extracts.

Arranged as it is, it forms a valuable book of reference to find out the nature of any remedy which the practitioner may never have heard of before, or the knowledge of which is insufficient. A book of this character is absolutely necessary for every intelligent physician, as it is not possible for him to keep up his knowledge of the myriads of new remedies that are introduced to the notice of the profession.

The editor of this journal is glad to have this book on his own shelves, and he has no hesitation in saying that every other doctor should have one.

